# Factors Associated with Recommendation of Non-Routine Childhood Vaccines Among Healthcare Workers in Türkiye

**DOI:** 10.3390/vaccines14080670

**Published:** 2026-08-02

**Authors:** Asiye Burcu Kuş, Adnan Barutçu, Sena Kara Öncü

**Affiliations:** Department of Pediatrics, Faculty of Medicine, Çukurova University, Adana 01330, Turkey; adnan_barutcu@hotmail.com (A.B.); senakaraoncu@hotmail.com (S.K.Ö.)

**Keywords:** healthcare workers, vaccine hesitancy, non-routine childhood vaccines, rotavirus, meningococcal vaccine, influenza vaccine, HPV vaccine, vaccination attitudes, Türkiye

## Abstract

**Background:** Healthcare workers play a critical role in parental vaccine decision-making and the uptake of non-routine childhood vaccines. This study aimed to evaluate healthcare workers’ knowledge, attitudes and behaviors regarding non-routine childhood vaccines, including rotavirus, meningococcal, human papillomavirus (HPV) and influenza vaccines. **Methods:** This cross-sectional study was conducted between October 2025 and March 2026 among healthcare workers in Türkiye. Data were collected using a structured questionnaire and the Attitudes Towards Vaccine Scale (ATVS), distributed through convenience and snowball sampling methods. **Results:** A total of 345 healthcare workers participated in the study. Recommendation behaviors differed significantly according to vaccine type (*p* < 0.001). Rotavirus and meningococcal vaccines were more frequently recommended than HPV and influenza vaccines. Although influenza vaccine had the highest awareness rate, it demonstrated the lowest recommendation rate and the lowest support for inclusion in the national immunization schedule. Physicians had significantly higher knowledge levels and vaccination attitude scores than non-physician healthcare workers (*p* < 0.001). ATVS scores were positively correlated with recommendation behaviors (ρ = 0.35, *p* < 0.001) and self-vaccination tendency (ρ = 0.34, *p* < 0.001). In multivariable analyses, higher vaccination attitude scores independently predicted greater self-vaccination tendency (OR = 1.07, 95% CI: 1.04–1.11, *p* < 0.001). Cost was the most commonly reported barrier to vaccine implementation. **Conclusions:** Positive attitudes toward non-routine childhood vaccines among healthcare workers do not necessarily translate into consistent recommendation behaviors across all vaccine types. Strategies targeting healthcare worker education, equitable vaccine access and standardized vaccine counseling practices may improve uptake and recommendation of optional childhood vaccines in Türkiye.

## 1. Introduction

Vaccination remains one of the most effective and cost-efficient public health interventions for preventing infectious diseases and reducing morbidity and mortality worldwide [1,2]. Despite substantial progress in global immunization programs, vaccine uptake remains suboptimal for several recommended and optional vaccines. According to World Health Organization estimates, approximately 14.3 million children remained completely unvaccinated in 2024, highlighting ongoing challenges in vaccine access and acceptance [1].

In Türkiye, childhood immunization services are implemented through the National Expanded Immunization Program, achieving high coverage rates for many vaccine-preventable diseases. However, several vaccines including rotavirus, meningococcal, HPV and seasonal influenza vaccines are not included in the national childhood immunization schedule and are mainly administered through individual access and out-of-pocket payment [3].

Healthcare workers play a central role in parental vaccine decision-making and are considered one of the most trusted sources of vaccine-related information. International evidence suggests that healthcare workers’ knowledge, vaccine confidence, attitudes and personal vaccination behaviors are associated with vaccine recommendation practices and parental vaccine acceptance. However, recommendation behaviors may vary across vaccine types and healthcare settings [4,5].

Although several Turkish studies have evaluated healthcare professionals’ knowledge or attitudes regarding selected non-routine childhood vaccines, studies simultaneously examining vaccine attitudes, recommendation practices, self-vaccination tendencies and multiple optional childhood vaccines across different healthcare professions remain limited [6,7,8].

Therefore, this study aimed to evaluate healthcare workers’ knowledge, general vaccination attitudes and recommendation behaviors regarding non-routine childhood vaccines. In addition, the study examined factors associated with general vaccination attitudes and self-vaccination tendency, as well as opinions regarding the inclusion of these vaccines in the National Immunization Program.

## 2. Methods

### 2.1. Study Design and Participants

This cross-sectional descriptive study was conducted between October 2025 and March 2026 among healthcare workers employed in various healthcare institutions across Türkiye.

Participants were recruited using convenience and snowball sampling methods through professional social media groups, institutional messaging networks and digital healthcare communication platforms. The survey was administered online. Given the voluntary online recruitment strategy, participation may have been influenced by individual interest in vaccination-related issues. Consequently, the study sample may not fully reflect the demographic characteristics of the overall healthcare workforce in Türkiye and certain professional or demographic groups may have been overrepresented.

Eligibility criteria included being aged 18 years or older, actively working as a healthcare professional in Türkiye and completing the survey in its entirety. Participation was voluntary and electronic informed consent was obtained from all participants before enrollment.

A total of 345 healthcare workers participated in the study. The adequacy of the sample size for the planned multivariable analyses was evaluated using G*Power version 3.1 [9]. Assuming a medium effect size (f^2^ = 0.15), a significance level of α = 0.05, a statistical power of 80% and a maximum of 10 predictors, the required minimum sample size was 118 participants [9]. Therefore, the final sample exceeded the required sample size and was considered adequate for the planned multivariable analyses. In addition, the events-per-variable criterion (EPV ≥ 10) was satisfied for the logistic regression analysis [10].

### 2.2. Data Collection Tools

Study data were collected using a structured online questionnaire developed by the researchers and the ATVS. The questionnaire was developed following a comprehensive review of the literature on non-routine childhood vaccines, healthcare workers’ vaccination attitudes, and vaccine recommendation practices. It included items assessing demographic and professional characteristics, awareness of non-routine childhood vaccines, recommendation behaviors, willingness to vaccinate one’s own child or relatives, opinions regarding the inclusion of vaccines in the National Immunization Program, perceived knowledge, sources of vaccine-related information and perceived barriers to vaccine implementation. Before the main study, the draft questionnaire was pilot-tested with 20 healthcare workers from different professional backgrounds to evaluate its clarity, comprehensibility and feasibility. Participants were asked to report any difficulties in understanding the questions, interpreting the response options or completing the questionnaire. Based on their feedback, minor wording modifications were made to improve clarity without altering the content or structure of the questionnaire. Data collected during the pilot phase were excluded from the final analyses.

The questionnaire included dichotomous (yes/no), multiple-choice and Likert-type items, depending on the variable being assessed. For statistical analyses, dichotomous variables were coded as 0/1, categorical variables according to their response categories and Likert-type responses were analyzed according to their original scale coding. The total ATVS score was calculated following the original scoring instructions of the validated instrument. The survey was administered using Google Forms and the “Limit to 1 response” setting was enabled to prevent duplicate submissions.

### 2.3. Attitudes Towards Vaccine Scale (ATVS)

Vaccination attitudes were assessed using the Attitudes Towards Vaccine Scale, developed by Cvjetkovic et al. [11]. The scale consists of 14 items rated on a five-point Likert scale ranging from 1 (“strongly disagree”) to 5 (“strongly agree”).

Negatively worded items were reverse-coded prior to analysis. Total scores range from 14 to 70, with higher scores indicating more positive general attitudes toward vaccination rather than attitudes toward any specific vaccine. Vaccine-specific recommendation behaviors were evaluated separately using the structured questionnaire.

For descriptive purposes, total scores were categorized as follows.

• 14–32 points: negative attitude.

• 33–51 points: moderate attitude.

• 52–70 points: positive attitude.

The Turkish validity and reliability study of the scale was conducted by Özümit and Yıldırım-Sarı. In the present study, internal consistency reliability was assessed using Cronbach’s alpha coefficient, which was calculated as 0.89 [12].

### 2.4. Ethical Approval

Ethical approval was obtained from the Ethics Committee of Çukurova University Faculty of Medicine (Meeting No: 158, Decision No: 17, Date: 12 September 2025).

Participation was voluntary and electronic informed consent was obtained from all participants before completion of the survey. The study was conducted in accordance with the ethical principles of the Declaration of Helsinki.

### 2.5. Statistical Analysis

Data were analyzed using IBM SPSS Statistics version 26.0 (IBM Corp., Armonk, NY, USA).

Descriptive statistics were used to summarize participants’ demographic and professional characteristics, awareness of non-routine childhood vaccines, recommendation behaviors, self-vaccination tendencies, opinions regarding inclusion of vaccines in the National Immunization Program, perceived knowledge adequacy, information sources and implementation barriers. Continuous variables were expressed as mean ± standard deviation (SD), minimum and maximum values, whereas categorical variables were presented as frequencies and percentages.

The normality of continuous variables was assessed using histograms, Q–Q plots and the Kolmogorov–Smirnov test. ATVS scores were analyzed both as continuous variables and according to predefined attitude categories.

Independent samples *t*-tests were used to compare ATVS scores between two groups. Comparisons involving more than two groups were performed using one-way analysis of variance (ANOVA). When significant differences were identified, Bonferroni-adjusted post hoc analyses were conducted to determine the source of the differences. Bonferroni adjustment was applied only to post hoc pairwise comparisons following statistically significant overall analyses. No multiple-testing correction was applied to the remaining statistical analyses.

Associations between categorical variables were examined using Chi-square tests. These analyses were used to compare vaccine awareness according to profession and workplace, as well as differences in recommendation frequency, willingness to vaccinate one’s own child or relatives, support for inclusion in the National Immunization Program and perceived knowledge adequacy across vaccine types.

Spearman correlation analysis was performed to evaluate relationships between ATVS scores, vaccine recommendation behaviors, self-vaccination tendency, support for inclusion in the National Immunization Program and perceived knowledge adequacy.

Multiple linear regression analysis was conducted to identify independent predictors of ATVS scores. Logistic regression analysis was performed to determine factors independently associated with self-vaccination tendency. Results were reported as regression coefficients (B), standardized beta coefficients (β), odds ratios (OR), 95% confidence intervals (CI) and *p*-values. For the regression analyses, physician status was operationalized as a binary variable. Specialist physicians, general practitioners, resident physicians and faculty members were classified as physicians whereas nurses, midwives and emergency medical technicians/paramedics were classified as non-physician healthcare workers.

Assumptions of the multiple linear regression model, including normality of residuals, homoscedasticity, linearity, multicollinearity, independence of residuals and influential observations were evaluated before model interpretation. For the logistic regression model, calibration was assessed using the Hosmer–Lemeshow goodness-of-fit test, whereas discrimination was evaluated using the area under the receiver operating characteristic (ROC) curve (AUC).

All statistical tests were two-tailed and a *p*-value < 0.05 was considered statistically significant.

## 3. Results

### 3.1. Participant Characteristics

A total of 345 healthcare workers participated in the study. Most participants were female (72.2%), employed in medical specialties (67.0%) and had children (67.8%). Nurses (35.4%) and specialist physicians (30.7%) constituted the largest professional groups. Detailed demographic and professional characteristics are presented in Table 1.

### 3.2. Awareness of Non-Routine Childhood Vaccines

Awareness of non-routine childhood vaccines was high across all vaccine types, ranging from 91.9% for meningococcal vaccine to 99.7% for influenza vaccine. Significant differences in vaccine awareness according to profession were observed only for meningococcal vaccine (χ^2^ = 62.97, *p* < 0.001; Cramér’s V = 0.427). Nearly all physicians (99%) were aware of the meningococcal vaccine, whereas awareness was substantially lower among paramedics/emergency medical technicians (74.2%). Similarly, meningococcal vaccine awareness differed significantly according to workplace (χ^2^ = 13.82, *p* = 0.032; Cramér’s V = 0.200). Awareness reached 100% among healthcare workers employed in Family Health Centers, Training and Research Hospitals, while comparatively lower rates were observed among employees of state hospitals (86.9%) and private hospitals (87.7%) (Table 2).

### 3.3. Vaccine Recommendation Practices and Attitudes Toward Non-Routine Vaccines

Healthcare workers’ recommendation behaviors differed significantly according to vaccine type (*p* < 0.001). Rotavirus and meningococcal vaccines were the most frequently recommended vaccines, whereas recommendation rates were substantially lower for HPV and influenza vaccines. Similar differences were observed regarding willingness to vaccinate one’s own child or relatives, support for inclusion in the National Immunization Program and perceived knowledge adequacy (Table 3).

Pairwise comparisons confirmed significantly higher recommendation rates and personal acceptance for rotavirus and meningococcal vaccines compared with HPV and influenza vaccines (all Bonferroni-adjusted *p* < 0.001). Detailed pairwise comparisons are presented in Table 4.

### 3.4. Vaccination Attitudes According to Professional Characteristics

The mean ATVS score was 57.89 ± 8.62 and 76.2% of participants were classified as having positive general vaccination attitudes. No significant difference in ATVS scores was observed between female and male healthcare workers (57.64 ± 8.73 vs. 58.54 ± 8.32, *p* = 0.386). ATVS scores differed significantly according to age group, profession, medical department and workplace (all *p* < 0.05). Post hoc analyses showed that participants aged 30–36 years had significantly higher ATVS scores than those aged 23–29 years (mean difference = 3.84, *p* = 0.030) and 44–50 years (mean difference = 4.81, *p* = 0.006). Regarding profession, nurses demonstrated significantly lower ATVS scores than specialist physicians, general practitioners and resident physicians (all adjusted *p* < 0.001). In addition, significant differences were observed between specialist physicians, paramedics/emergency medical technicians and midwives. Participants working in Medical Sciences had significantly higher ATVS scores than those in Surgical Sciences (mean difference = 5.42, *p* < 0.001) and Basic Medical Sciences (mean difference = 5.01, *p* = 0.027), whereas no significant difference was found between Surgical and Basic Medical Sciences (*p* = 1.000).

### 3.5. Correlations Between Attitudes, Knowledge and Behaviors

ATVS scores were positively correlated with all behavioral indicators, including recommendation frequency (ρ = 0.35), self-vaccination tendency (ρ = 0.34), support for inclusion in the National Immunization Program (ρ = 0.24), perceived knowledge adequacy (ρ = 0.24) and vaccine awareness (ρ = 0.23) (all *p* < 0.001). The strongest association was observed between self-vaccination tendency and support for inclusion in the National Immunization Program (ρ = 0.56, *p* < 0.001) (Table 5).

### 3.6. Multivariable Analyses

Being a physician and higher recommendation frequency were independently associated with higher ATVS scores. Higher ATVS scores independently predicted greater self-vaccination tendency, whereas male sex and having children were associated with lower odds of self-vaccination. The logistic regression model showed good calibration based on the Hosmer–Lemeshow goodness-of-fit test (*p* = 0.807) and acceptable discrimination (AUC = 0.723, 95% CI: 0.670–0.776). Detailed regression results are presented in Table 6 and Table 7.

### 3.7. Information Sources and Perceived Barriers

The most frequently reported information sources were medical education/training (35.7%) and Ministry of Health guidelines (26.4%). Cost was identified as the most commonly reported barrier to non-routine vaccine implementation (39.7%).

## 4. Discussion

This study demonstrated that healthcare workers do not evaluate all non-routine childhood vaccines similarly. Although awareness levels were consistently high across all vaccine types, recommendation behaviors, personal vaccination tendencies and support for inclusion in the National Immunization Program varied substantially. These findings suggest that healthcare workers’ vaccine-related behaviors may be associated not only with knowledge but also with vaccine-specific perceptions and contextual factors. Importantly, the present findings indicate that healthcare workers should not simply be categorized as having either positive or negative vaccination attitudes. Although overall vaccination attitudes were generally favorable, recommendation behaviors differed substantially across vaccine types. This pattern is more consistent with selective vaccine acceptance than with generalized vaccine hesitancy, suggesting that healthcare workers evaluate individual vaccines according to their perceived benefits, disease burden, expected effectiveness, accessibility and broader contextual factors.

One of the most notable findings was the discrepancy observed for influenza vaccination. Despite having the highest awareness rate among healthcare workers, influenza vaccine demonstrated the lowest recommendation rate and the lowest level of support for inclusion in the National Immunization Schedule. This finding suggests that awareness alone may be insufficient to promote recommendation behavior [13,14]. Several factors may contribute to this attitude–behavior gap. Influenza is often perceived as a relatively mild disease compared with other vaccine-preventable infections, potentially reducing the perceived necessity of routine vaccination. In addition, the requirement for annual revaccination and the variable effectiveness of seasonal influenza vaccines may weaken confidence in recommending the vaccine. Another possible explanation is that influenza vaccination is frequently viewed as an adult or high-risk group vaccine rather than a routine childhood immunization. Similar patterns have been reported in previous studies demonstrating that healthcare workers’ influenza vaccine recommendations are influenced by perceived disease severity, vaccine effectiveness and professional norms rather than knowledge alone [15,16]. In addition, national vaccination policies in Türkiye prioritize influenza vaccination primarily for selected risk groups rather than universal childhood immunization. This policy context may also influence healthcare workers’ perceptions regarding the relative priority of recommending influenza vaccination for healthy children.

The findings regarding HPV vaccination are also noteworthy. Compared with rotavirus and meningococcal vaccines, HPV vaccine demonstrated lower recommendation rates, lower willingness to vaccinate one’s own child or relatives and lower support for inclusion in the National Immunization Schedule. Several contextual factors may explain these findings. In Türkiye, HPV vaccine is not included in the routine immunization program and requires out-of-pocket payment, which may negatively influence both acceptance and recommendation behaviors [17]. Furthermore, HPV infection is frequently associated primarily with sexual transmission and cultural sensitivities surrounding sexuality may affect vaccine discussions with parents and adolescents. Limited awareness of the broader benefits of HPV vaccination, including protection against multiple HPV-related cancers and its importance for both females and males may also contribute to lower recommendation rates. Previous studies have similarly identified cost concerns, cultural perceptions and knowledge gaps as important barriers to HPV vaccine acceptance and recommendation [18,19].

Professional differences represented another important finding of this study. Physicians demonstrated more positive vaccination attitudes than non-physician healthcare workers. These differences may reflect variations in immunization training, clinical exposure and involvement in vaccine counseling. Physicians receive more extensive education regarding infectious diseases, preventive medicine and immunization practices, and are more frequently involved in discussions with families regarding vaccination decisions. Such experiences may contribute to greater confidence in vaccine safety and effectiveness. Importantly, the positive association observed between ATVS scores and recommendation behaviors suggests that healthcare workers’ personal confidence in vaccines may directly influence their clinical practices. This finding is consistent with previous studies showing that vaccine confidence is an important determinant of both personal vaccination behavior and vaccine recommendation practices [19,20]. Therefore, interventions aimed at improving recommendation behaviors should focus not only on increasing knowledge but also on strengthening trust and confidence in vaccination [21,22].

Cost emerged as the most frequently reported barrier to non-routine vaccine implementation. This finding highlights the importance of structural determinants of vaccination behavior. In healthcare systems where optional vaccines require direct out-of-pocket payment, healthcare workers may be less likely to recommend vaccines that they perceive as financially inaccessible to families [23,24]. Similar associations between vaccine cost and recommendation practices have been reported in previous studies evaluating optional childhood vaccines [25,26].

Taken together, these findings suggest that favorable general vaccination attitudes do not necessarily translate into uniform recommendation behaviors across all vaccines. Rather than reflecting generalized vaccine hesitancy, healthcare workers appeared to demonstrate selective vaccine acceptance, recommending some non-routine childhood vaccines more consistently than others. This pattern likely reflects differences in perceived disease severity, expected vaccine effectiveness, cost, accessibility and sociocultural context.

Although female healthcare workers were overrepresented in our study sample (72.2%), women also represent a substantial proportion of the healthcare workforce in Türkiye. According to national statistics published by the Turkish Ministry of Health, women comprise approximately 57.4% of the healthcare workforce, with considerably higher representation among nurses and midwives than among physicians [27]. Therefore, the predominance of female participants in our study may partly reflect the professional composition of the recruited sample rather than recruitment bias alone. Nevertheless, because participation was voluntary and recruitment was conducted online, some degree of selection bias cannot be excluded. Importantly, no significant difference in general vaccination attitude scores was observed between female and male healthcare workers, suggesting that the overrepresentation of women was unlikely to have materially influenced the overall findings.

This study has several limitations. Due to the cross-sectional design, causal relationships cannot be established. In addition, the use of convenience and snowball sampling may have introduced selection bias, as healthcare workers with more positive attitudes toward vaccination may have been more likely to participate. Consequently, vaccine awareness, general vaccination attitude scores and vaccine recommendation behaviors observed in this study may have been overestimated compared with those of the overall healthcare workforce in Türkiye, and the professional distribution of the study sample was not fully representative of the national healthcare workforce composition in Türkiye [27]. Furthermore, because the study relied on self-reported data, reporting bias and social desirability bias cannot be excluded, particularly regarding self-vaccination tendency and vaccine recommendation behaviors. Moreover, the questionnaire was not designed to investigate the underlying reasons for vaccine-specific recommendation preferences or to evaluate potentially relevant contextual factors, such as previous vaccine training, frequency of pediatric practice, exposure to vaccine misinformation, personal vaccination history and institutional vaccination policies. Future studies incorporating these variables may provide a more comprehensive understanding of healthcare workers’ vaccine recommendation behaviors. Therefore, the findings should be interpreted with caution when generalizing to all healthcare workers in Türkiye.

## 5. Policy Implications

The findings of this study suggest that strategies aimed at improving the uptake of non-routine childhood vaccines should extend beyond increasing awareness alone. The observed differences in recommendation behaviors across vaccine types, despite generally high awareness levels, suggest that vaccine-specific perceptions and attitudes may be associated with healthcare workers’ recommendation practices. Therefore, interventions designed to strengthen healthcare workers’ education and training on non-routine childhood vaccines, reinforce confidence in vaccine safety and effectiveness and improve communication skills, particularly among professional groups demonstrating lower ATVS scores, may help promote more consistent recommendation behaviors across healthcare settings.

The identification of cost as the most frequently reported barrier further highlights the importance of structural determinants in vaccine-related decision-making. In healthcare systems where non-routine vaccines are primarily financed through out-of-pocket payments, financial considerations may influence both vaccine uptake and recommendation practices. Policies aimed at improving affordability and evaluating broader access to selected non-routine vaccines may contribute to reducing inequalities in access and supporting more equitable implementation of childhood immunization services in Türkiye. Reducing economic barriers may not only improve vaccine uptake among families but also increase healthcare workers’ confidence in recommending these vaccines [28]. Furthermore, the high level of support among healthcare workers for the inclusion of selected non-routine childhood vaccines in the National Immunization Program suggests that expanding the national immunization schedule may represent an important policy strategy to promote more consistent vaccine recommendation practices and improve vaccine uptake in Türkiye.

## 6. Conclusions

This study demonstrated that healthcare workers’ knowledge, attitudes and behaviors regarding non-routine childhood vaccines are closely related but do not always translate into consistent recommendation practices. Although awareness levels were high across all vaccine types, recommendation behaviors varied substantially, with lower recommendation rates observed particularly for influenza and HPV vaccines. The findings further indicate that healthcare workers may demonstrate selective rather than uniform positive vaccination attitudes, recommending certain non-routine childhood vaccines more consistently than others despite generally favorable overall vaccination attitudes. Positive general vaccination attitudes were associated with greater recommendation frequency and stronger self-vaccination tendencies, suggesting that personal vaccine confidence may be reflected in professional recommendation practices.

Cost emerged as the most commonly reported barrier to non-routine vaccine implementation, suggesting that structural and economic factors may affect vaccine-related behaviors beyond knowledge alone. Educational interventions targeting healthcare workers, standardized vaccine counseling practices and policies aimed at reducing financial barriers may contribute to more consistent vaccine recommendations and improved uptake of non-routine childhood vaccines in Türkiye.

## Figures and Tables

**Table 1:** Demographic Characteristics of Participants.

Variable	Category	*n*	%
**Sex**	Female	249	72.2
	Male	96	27.8
**Age group**	23–29 years	79	22.9
	30–36 years	96	27.8
	37–43 years	91	26.4
	44–50 years	48	13.9
	≥51 years	31	9.0
**Profession**	Nurse	122	35.4
	Specialist physician	106	30.7
	Paramedic/Emergency Medical Technician (EMT)	33	9.5
	General practitioner	27	7.8
	Resident physician	25	7.2
	Midwife	21	6.0
	Faculty member	11	3.1
**Medical department**	Medical Sciences (Internal Medicine)	231	67.0
	Surgical Sciences	94	27.2
	Basic Medical Sciences	20	5.8
**Workplace**	State hospital	99	28.7
	University hospital	95	27.5
	Private hospital	81	23.5
	Family Health Center	32	9.3
	Training and Research Hospital	22	6.4
	Other/City hospital	16	4.6
**Professional experience**	1–8 years	120	34.9
	9–16 years	129	37.5
	17–24 years	54	15.7
	25–32 years	29	8.4
	≥33 years	13	3.8
**Having children**	Yes	234	67.8
	No	111	32.2

**Table 2:** Awareness of Non-Routine Childhood Vaccines According to Profession and Workplace.

Vaccine	Overall Awareness *n* (%)	Profession χ^2^	*p*	Cramér’s V	Workplace χ^2^	*p*	Cramér’s V
**Rotavirus**	330 (95.7)	11.38	0.329	0.182	10.60	0.102	0.175
**Meningococcal**	317 (91.9)	62.97	<0.001	0.427	13.82	0.032	0.200
**HPV**	333 (96.5)	8.38	0.591	0.156	6.87	0.333	0.141
**Influenza**	344 (99.7)	NA	NA	NA	NA	NA	NA

HPV: human papillomavirus; χ^2^: chi-square statistic; Cramér’s V: effect size for chi-square tests; NA: not applicable.

**Table 3:** Recommendation Practices, Personal Vaccination Tendency, Support for Inclusion in the National Immunization Program and Perceived Knowledge According to Vaccine Type.

Variable	Rotavirus *n* (%)	Meningococcal *n* (%)	HPV *n* (%)	Influenza *n* (%)	χ^2^	*p*
Always recommends	210 (60.9)	209 (60.6)	159 (46.1)	132 (38.3)	85.33	<0.001
Vaccinated/willing to vaccinate	267 (77.4)	266 (77.1)	217 (62.9)	192 (55.7)	56.21	<0.001
Should be included in national schedule	282 (81.7)	308 (89.3)	264 (76.5)	169 (49.0)	170.73	<0.001
Feels knowledgeable	254 (73.6)	229 (66.4)	222 (64.3)	264 (76.5)	18.08	0.006

HPV: Human papilloma virus.

**Table 4:** Pairwise Comparisons Between Vaccine Types for Behavioral Indicators.

Behavioral Indicator	Comparison	χ^2^	*p*
Always recommends	Rotavirus vs. HPV	24.27	<0.001
Always recommends	Rotavirus vs. Influenza	52.94	<0.001
Always recommends	HPV vs. Influenza	8.14	0.004
Personal vaccination	Rotavirus vs. HPV	23.09	<0.001
National schedule support	Meningococcal vs. Influenza	115.42	<0.001

HPV: Human papilloma virus.

**Table 5:** Correlations Between Vaccination Attitudes, Knowledge and Behavioral Indicators.

Variable	1	2	3	4	5	6
1. ATVS score	—					
2. Recommendation frequency	0.35 **	—				
3. Vaccination tendency	0.34 **	0.48 **	—			
4. National schedule support	0.24 **	0.38 **	0.56 **	—		
5. Perceived knowledge	0.24 **	0.34 **	0.22 **	0.22 **	—	
6. Vaccine awareness	0.23 **	0.26 **	0.16 *	0.09	0.29 **	—

ATVS: Attitudes Towards Vaccine Scale; Spearman correlation analysis * all *p* < 0.05; ** all *p* < 0.001.

**Table 6:** Multiple Linear Regression Analysis of Factors Associated with General Vaccination Attitudes (ATVS Score).

Predictor	B	SE	Standardized β	95% CI	*p*
**Physician status**	7.97	0.84	0.926	6.32– 9.61	<0.001
**Recommendation frequency**	1.28	0.47	0.140	0.36–2.20	0.006

B: unstandardized regression coefficient; SE: standard error; β: standardized regression coefficient; CI: confidence interval.

**Table 7:** Multivariable Logistic Regression Analysis of Factors Associated with Self-Vaccination Tendency.

Predictor	B	SE	Wald χ^2^	OR	95% CI	*p*
**ATVS score**	0.070	0.017	16.52	1.07	1.04–1.11	<0.001
**Male sex**	−0.677	0.286	5.58	0.51	0.29–0.89	0.018
**Having children**	−0.736	0.309	5.67	0.48	0.26–0.88	0.017

ATVS: Attitudes Towards Vaccine Scale; B: regression coefficient; SE: standard error; OR: odds ratio; CI: confidence interval.

## Data Availability

The data presented in this study are available from the corresponding author upon reasonable request.
